# New Appliances and Things Medical

**Published:** 1911-02-18

**Authors:** 


### NEW APPLIANCES & THINGS MEDICAL.

[We shall be glad to receive at our Office, 28 & 29 Southampton Street, Strand, London, W.C., from the manufacturers, specimens of all new preparations and appliances.]

### "SULIS WATER."

(CATER, STOFFELL & FORTT, LTD., THE SPRINGS BATH.)

THE samples of this table water which have been submitted to us proved to be most interesting from many points of view. To begin with, "Sulis" is an aerated English spa water. It is obtained from the Sulis Springs at Bath—of which an account was published in THE HOSPITAL of January—which were known to the ancient Romans, and which yield a mild chalybeate water of the finest quality. Analysis proves this water, in its aerated state as supplied to us, to contain a relatively large amount of sulphates of calcium and soda, with smaller percentages of the chlorides of magnesium and sodium, and still smaller percentages of sulphate of potash and bicarbonate of lime. In addition it contains traces of bicarbonate of magnesium and of the nitrates of ammonia and calcium, with more than one per cent. of carbonate of iron. Its total solids per gallon average 169. In appearance it is remarkably sparkling, clear, and "springy" water, which mixes admirably with wine and spirit or syrups, and which possesses a crisp flavour of its own which, in our opinion, surpasses that of most table waters on the market. It is a mild tonic and aperient, and as a table water it is undoubtedly one of the finest procurable. Infinite care is taken in preparing and aerating it, and every bottle that was opened was found to be of uniform flavour and sparkle. The price of this table water is very moderate indeed, and we feel sure that Sulis needs only to be known to become the most popular table drink for those who desire a pure unadulterated water, that can be used in its natural state or as an excipient. The amount of iron it contains, though too small to influence the taste to any perceptible degree, is undoubtedly of importance as a mineral tonic, and Sulis may therefore be recommended to those who require such and who find the ordinary ferruginous waters too metallic for their taste. The water is put up in neat bottles, half bottles, and splits.

### DIURETIN.

(MESSRS. KNOLL & CO., 8 HARP LANE, LONDON, E.C.)

THE double salt of theobromine sodium and sodium salicylate which Messrs. Knoll have put on the market under the name of "Diuretin" is a white amorphous powder, free from smell and with a slightly saline taste. It is readily soluble in water, and is rapidly absorbed when given by the mouth. Diuretin is practically without direct effect on the nervous system, and has proved a very valuable diuretic remedy and heart tonic. It is particularly recommended in cases of cardiac dropsy. Messrs. Knoll have just issued a neat little manual on the drug, which gives all the literature, together with full details with regard to dosage, method of combination, and indications for use, and which should prove very valuable to practitioners who desire to test the remedy. It is supplied post free on application to the firm at the above address.

